# Exogenous Glycine Betaine Decreases Cell Proliferation and Induces Apoptosis in Human Colorectal Adenocarcinoma HT-29 Cells

**DOI:** 10.3390/ijms262110751

**Published:** 2025-11-05

**Authors:** Lizeth López-Castro, Jesús Rosas-Rodríguez, Ramona Icedo-García, Norma Stephens-Camacho, Guadalupe Gonzalez-Ochoa

**Affiliations:** 1 Laboratorio de Microbiología e Inmunología, Departamento de Ciencias Químico-Biológicas y Agropecuarias, Facultad Interdiciplinaria de Ciencias Biológicas y de Salud, Universidad de Sonora, Blvd. Lázaro Cárdenas No. 100 Col. Francisco Villa, Navojoa 85880, Sonora, Mexico; nutrilizeth@gmail.com (L.L.-C.); jesus.rosas@unison.mx (J.R.-R.); ramona.icedo@unison.mx (R.I.-G.); 2 Licenciatura en Nutrición Humana, Universidad Estatal de Sonora, Blvd. Manlio Fabio Beltrones 810, Col. Bugambilias, Navojoa 85875, Sonora, Mexico

**Keywords:** glycine betaine, cancer, HT-29 cells, p53, caspase-3

## Abstract

Studies in cervical and prostate cancer cells have reported that frequent consumption of foods rich in glycine betaine (GB) and choline have beneficial effects against some types of cancer. However, the role of GB against the human colorectal adenocarcinoma cell line HT-29 has not yet been elucidated. Therefore, this study aimed to evaluate the effect of GB on p53 and caspase-3 expression, which regulate cellular processes such as cell proliferation and apoptosis, respectively, on HT-29 cells. HT-29 cells were treated with GB at 5 mg/mL, 15.6 mg/mL, 31.2 mg/mL, and 62.5 mg/mL, after which RNA purification and cDNA synthesis were performed, followed by qPCR to detect the relative expression of p53 and caspase-3, using GAPDH as a reference gene, and protein levels were determined by ELISA. Results indicated that in HT-29 cells treated with GB at 62.5 mg/mL, the protein levels of p53 significantly (*p* < 0.05) increased to 45 U/mL, as compared with cells without GB (21 U/mL), whereas caspase-3 increased to 30 ng/mL, as compared with control cells (20.13 ng/mL). Therefore, we conclude that GB at high concentrations decreases cell proliferation and induces apoptosis in HT-29 cells.

## 1. Introduction

Glycine betaine (GB) is synthesized in cells from choline and is obtained by food consumption [1,2]. GB is an osmolyte associated with cell protection under osmotic stress conditions [3]. It is also a significant methyl donor in the methionine cycle [4]. Moreover, supplementation with GB and food rich in GB have been related to anti-inflammatory activity in diseases such as diabetes, non-alcoholic fatty liver, and cancer [1,2].

In this regard, high intake of choline and GB has been associated with breast cancer prevention and with lower mortality after breast cancer diagnosis [5]. Furthermore, GB has been related to tumoricidal effects in the human cervical cancer cell line HeLa and the prostate cancer cell line DU-145 [6,7]. In addition, Seyyedsalehi and others reported the association between GB intake and a significant decrease in colorectal cancer (CRC) risk [8,9]. However, other studies do not support such an association [10,11].

In 2020, over 1.9 million new CRC cases and 930,000 deaths were estimated worldwide [12]. The management of CRC includes surgery and its combination with chemotherapy and/or radiotherapy [13]. These treatments have unfavorable side effects for patients such as loss of appetite, dental problems, changes in taste and smell, diarrhea, constipation, fatigue, and depression [14]. As a complementary strategy to cancer treatment, the European Society for Clinical Nutrition and Metabolism (ESPEN) suggests monitoring the nutritional status of patients and the nutritional quality of the foods they consume to promote a good prognosis for the disease and reduce the side effects of therapies [15,16]. Among these components is GB, which is found in a variety of foods, including mollusks, wheat, quinoa, spinach, and beets [17]. It may also be obtained from supplements [18]. GB has been shown to have effects against cancerous cells through the activation of genes such as p53, which is involved in proliferation and cell cycle regulation [7,19] and caspase-3, also known as effector caspase, responsible for inducing apoptosis or programmed cell death in in vitro studies [6,7,20,21].

To date, the effect of GB on the colon adenocarcinoma cell line HT-29 has not been yet elucidated. Therefore, the present study aimed to evaluate the effect of GB against HT-29 cells, using p53 and caspase-3 expression levels to determine the effect on proliferation and apoptosis, respectively.

## 2. Results

### 2.1. Cellular Viability and Proliferation

**Figure 1:**
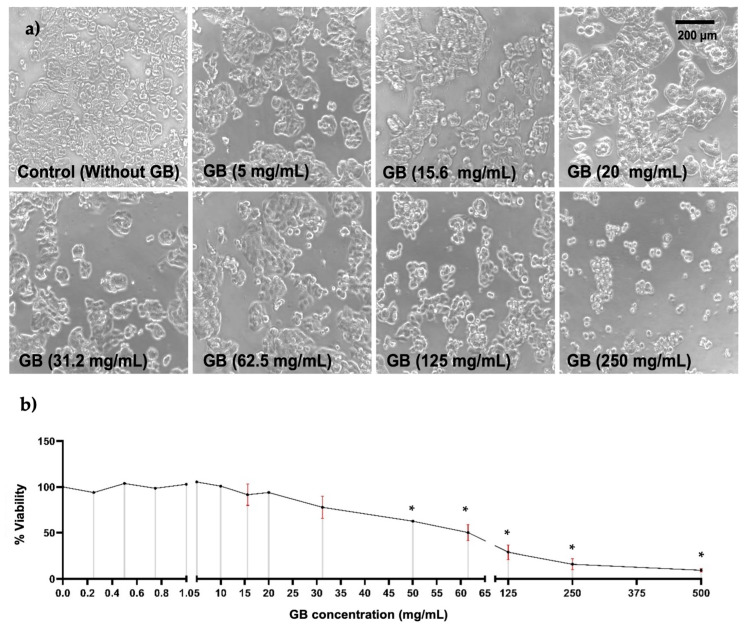
HT-29 cells viability. HT-29 cells were incubated for 24 h with various concentrations of GB. (**a**) Images of HT-29 cells incubated with GB observed under optical-inverted light microscopy, alterations in cells morphology such as smaller appearance, less refracted, rounded, contraction, and cellular blebbing were observed in cells treated with GB at concentrations above 31.2 mg/mL. Scale bar: 200 μm. (**b**) HT-29 cells viability after incubation with GB by the MTT reduction assay indicated 50% viability of cells treated with 62.5 mg/mL (*p* < 0.05). Data represents the mean of at least three determinations from three independent experiments. Once the normality of the data was confirmed, a one-way ANOVA test was performed, with a confidence level of *p* < 0.05, an asterisk (*) represents a statistically significant difference in comparison with HT-29 cells without GB. Key variable: GB concentration. Units of measure: mg/mL of GB and % of viability respect to control.

To study the effect of GB on HT-29 cells’ viability, they were incubated with GB at concentrations ranging from 0.2 mg/mL to 500 mg/mL for 24 h. Alterations in cellular morphology of HT-29 were observed in cells treated with GB at concentrations above 31.2 mg/mL; these alterations were smaller cellular appearance, less refracted, rounded, contraction, cellular blebbing, and damaging interactions with near cells (Figure 1a). Furthermore, results of cellular viability measured by the MTT reduction assay indicated a viability ranging from 91% to 100% of cells treated with GB at 0.25 mg/mL to 15.6 mg/mL, 78% viability of cells treated with 30 mg/mL GB, 50% viability of cells treated with 62.5 mg/mL GB, and 29%, 15%, and 9% viability of cells incubated with 125 mg/mL, 250 mg/mL, and 500 mg/mL GB, respectively (Figure 1b).

**Figure 2:**
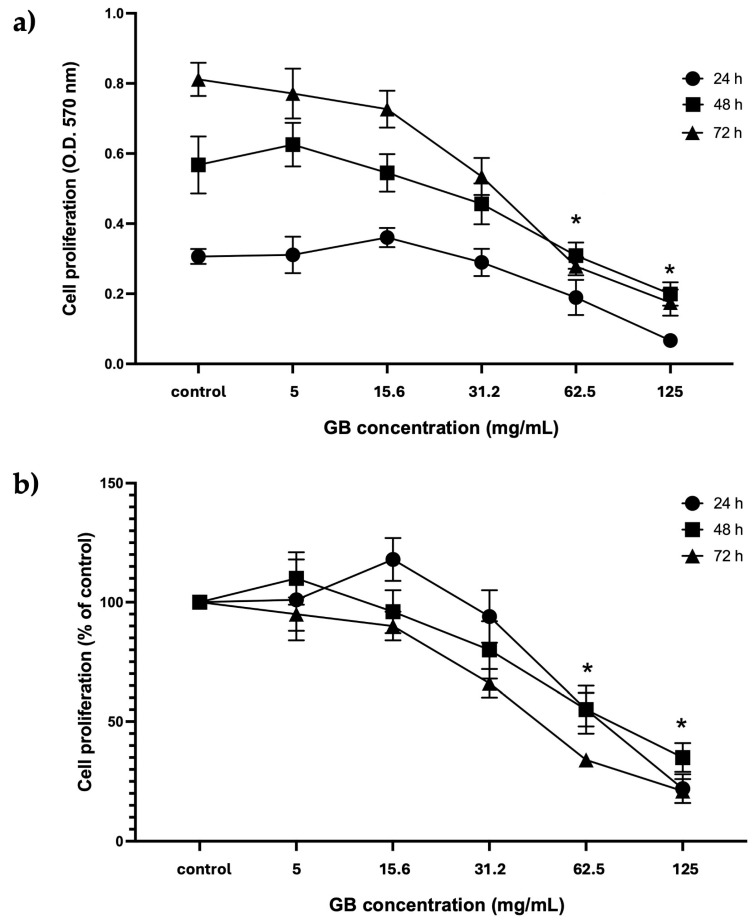
GB effect on HT-29 cells’ proliferation and viability. (**a**) GBs effect on cell proliferation (absorbance at 570 nm, which is proportional to cell number in the MTT assay). HT-29 cells were treated with 5 mg/mL to 125 mg/mL GB for 24 h, 48 h, and 72 h. (**b**) GB effect on viability (percentage of control). HT-29 cells were treated with 5 mg/mL to 125 mg/mL GB for 24 h, 48 h, and 72 h. Control cells were grown in a medium without GB. Data represents the mean ± SD of triplicate determinations from three independent experiments. An asterisk (*) represents a statistically significant difference (*p* < 0.05) in comparison with HT-29 cells without GB.

HT-29 cells treated with GB at concentrations from 5 mg/mL to 125 mg/mL were considered for further experiments. The proliferation of cells treated with GB was assessed using the colorimetric MTT reduction assay and reported as optical density (O.D.) values and as a percentage of control at 24 h, 48 h, and 72 h. Results of viability of HT-29 cells incubated with GB showed the highest absorbance at 570 nm, which is considered proportional to the cell number, in cells incubated 72 h with GB at 5 mg/mL and 15.6 mg/mL, as compared with cells incubated 24 h and 48 h at 5 mg/mL and 15.6 mg/mL GB. There was no significant difference between the O.D. of cells incubated with GB at concentrations of 5 mg/mL and 15.6 mg/mL for 24 h, 48 h, and 72 h, as compared with their controls (Figure 2a). In contrast, a significant (*p* < 0.05) reduction in absorbance (hence in cell number) was observed in cells treated with GB at 62.5 mg/mL and 125 mg/mL for 24 h, 48 h, and 72 h, as compared with the control without GB (Figure 2b). Moreover, cell viability (percentage of control) decreased to 40% and 55% at GB concentrations of 62.5 mg/mL and 125 mg/mL, respectively, at 24 h, 48 h, and 72 h (Figure 2b).

### 2.2. p53 Levels in GB-Treated HT-29 Cells

**Figure 3:**
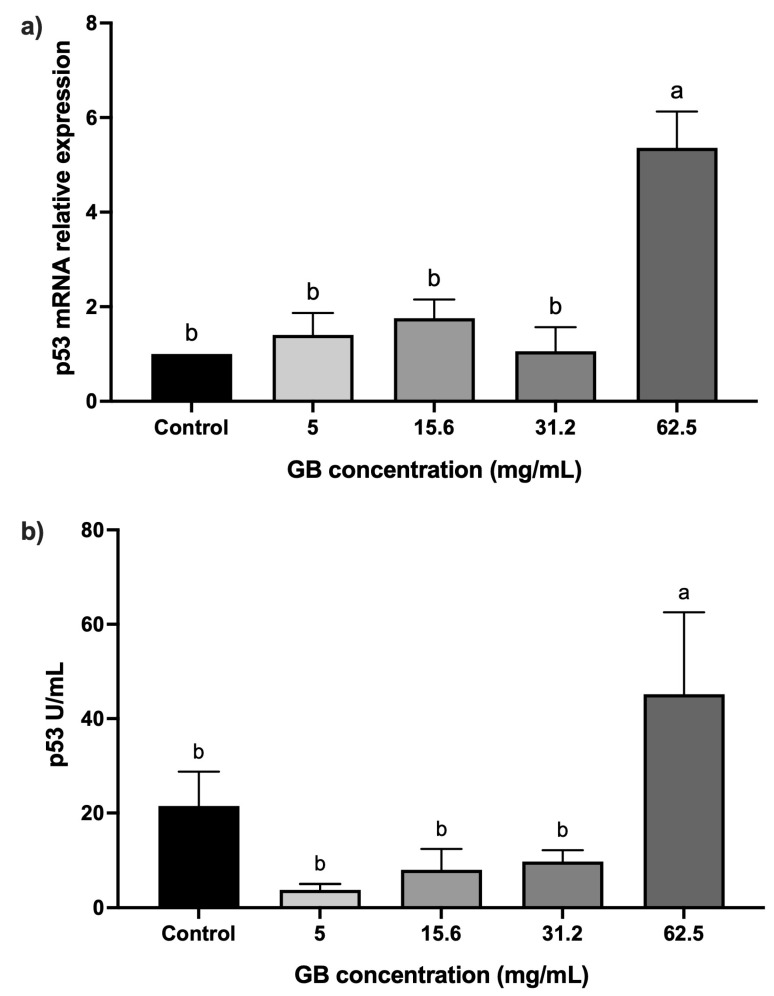
p53 levels in GB-treated HT-29 cells. (**a**) p53 mRNA relative expression levels in HT-29 cells incubated with GB (p53/GAPDH). (**b**) Soluble protein levels of p53 in HT-29 cells incubated with GB. Data represents the mean ± SD of triplicate determinations from three independent experiments. Different letters indicate statistical significance (*p* < 0.05) between treatments.

HT-29 cells were incubated with GB at 5 mg/mL, 15 mg/mL, 31.2 mg/mL, and 62.5 mg/mL) for 24 h, after which the relative expression and protein levels of p53 were determined. Cells incubated with GB at concentrations ranging from 5 mg/mL to 31.2 mg/mL did not have a statistically significant difference in p53 relative expression, as compared with the control, whereas in cells treated with 62.5 mg/mL GB, p53 relative expression increased fivefold (*p* < 0.05), as compared with the control (Figure 3a). In addition, p53 protein levels (45 U/mL) were significantly (*p* < 0.05) higher in cells treated with 62.5 mg/mL GB, as compared with the untreated control (21.45 U/mL) (Figure 3b).

### 2.3. Caspase-3 Levels in GB-Treated HT-29 Cells

**Figure 4:**
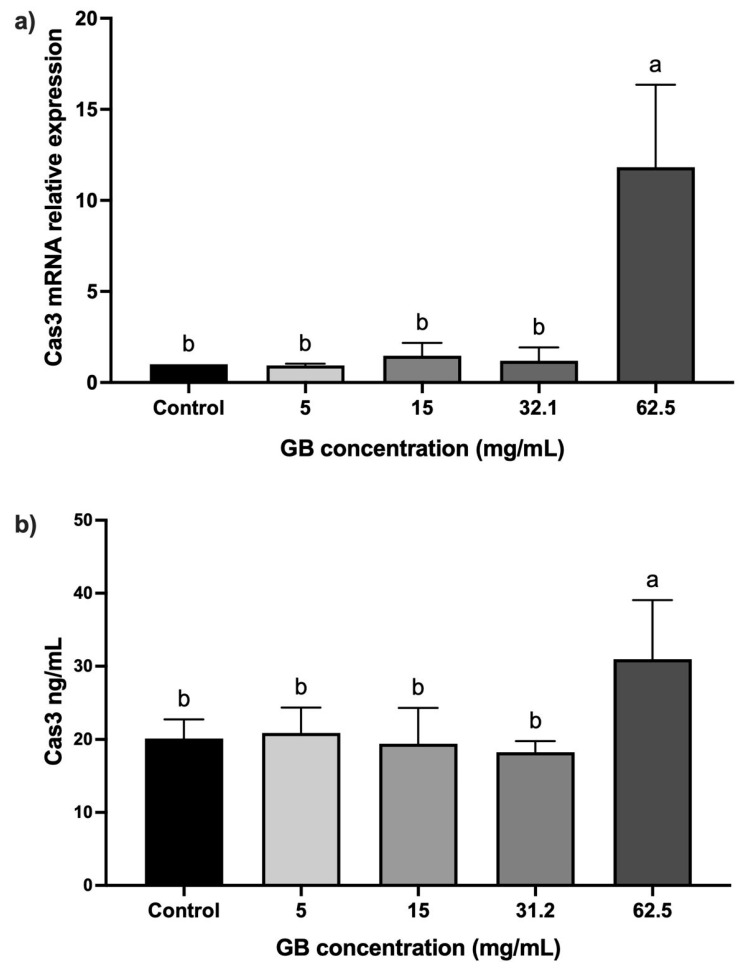
Caspase-3 levels in GB-treated HT-29 cells. (**a**) Caspase-3 mRNA relative expression levels in HT-29 cells incubated with GB (caspase-3/GAPDH), (**b**) soluble protein levels of caspase-3 in HT-29 cells treated with GB. Data represents the mean ± SD of triplicate determinations from three independent experiments. Different letters indicate statistical significance (*p* < 0.05) between treatments.

The mRNA relative expression and protein levels of caspase-3 were determined in HT-29 cells incubated with GB at 5 mg/mL, 15 mg/mL, 20 mg/mL, 31.2 mg/mL, and 62.5 mg/mL) for 24 h. In cells incubated with GB at concentrations between 5 mg/mL and 31.2 mg/mL, the caspase-3 mRNA relative expression was not significantly different, as compared with the control. In contrast, in cells treated with 62.5 mg/mL GB, we observed a significant (*p* < 0.05) 11-fold increase in the caspase-3 mRNA relative expression, as compared with the control (Figure 4a). A significant (*p* < 0.05) increase in caspase-3 (30 ng/mL) protein level was also observed in cells incubated with 62.5 mg/mL GB, as compared with the control (20 ng/mL) (Figure 4b).

## 3. Discussion

The potential therapeutic effects of GB consumption in humans have not been well described yet. Few clinical trials provided evidence that GB may decrease the risk of CRC [22]. In addition, doses ranging from 25 mg/kg/day to 150 mg/kg/day have shown to improve the response to chemotherapy in leukemia patients [23]. In the present study, we first determined the effect of GB on HT-29 cells’ viability. These cells were incubated with GB at various concentrations, based on previous studies reporting its use at low and high concentrations to study anti-cancer properties [6,7,24]. HT-29 cells incubated with GB at concentrations of 31.2 mg/mL and 62.5 mg/mL for 24 h showed morphology alterations and the presence of cellular debris (Figure 1a), and cellular viability dropped to 78% and 50%, respectively. In this regard, previous research reported that GB concentrations higher than 5 mg/mL in HeLa cells were associated with changes in morphology and membrane blebbing [7].

GB-induced HT-29 cells proliferation was assessed using the MTT reduction assay and reported as O.D. values and as a percentage of control at 24 h, 48 h, and 72 h. We observed that cellular proliferation and viability significantly decreased in cells incubated with GB at concentrations of 62.5 mg/mL or higher. Previous reports showed a dose-dependent effect of GB in inhibiting cellular proliferation and decreasing viability in cancer cell lines; in HeLa cells, proliferation decreased by 40% to 80%, and in HepG2 cells, it decreased by 25% [7,25]. In addition, in the human cells oral squamous cell carcinoma cell lines HSC-4 and HSC-7, GB effectively suppressed cellular proliferation, triggered early apoptosis, and reduced cell migration [26].

To determine if cell viability and proliferation were associated with p53 and caspase-3 expression in GB-treated cells, we evaluated p53 and caspase-3 protein relative expression in HT-29 cells. Cells incubated with GB at concentrations between 5 mg/mL and 31.2 mg/mL did not show significant differences, as compared with untreated cells. Nevertheless, in cells incubated with GB at 62.5 mg/mL, p53 and caspase-3 levels were overexpressed compared with the control (*p* < 0.05). In this regard, previous studies reported an increased expression of p53 and caspase-3 in HeLa cells at concentrations of GB between 20 mg/mL and 100 mg/mL [7], and an increased expression level of caspase-3 was associated with cellular death in the human prostate cancer cell line DU-145 incubated with 50 mg/mL of GB [6]. Moreover, the use of whey (which is rich in GB) in HT-29 inhibited cell proliferation, and induced cell cycle arrest and apoptosis via caspase-3 activation [20]. Therefore, the anti-cancer effect of GB was associated with p53 activation, whereas caspase-3 activation induces cellular apoptosis [27]. Other studies have shown the effect of beetroot extract (rich in GB) on the human breast cancer MCF-7 cell line), showing an increase in apoptotic-related proteins, such as p53 [28].

In the present study, we aimed, as a first objective, to evaluate the effect of GB at various concentrations on HT-29 cells proliferation, without considering the use of anti-cancer drug controls (vincristine or cisplatin) as positive controls, because we previously reported that vincristine caused 60% HT-29 cell growth inhibition from 0.0037 μg/mL [29]. Moreover, although the HT-29 cells model to study human colon cancers is widely used, it fails to accurately mimic the in vivo growth characteristics of tumor cells [30].

Several studies in vitro and in vivo models support the therapeutic potential of GB as an anti-inflammatory and preventive agent in processes related to colorectal inflammation [22]. GB administration significantly reduced the incidence of tumor formation and the level of inflammation in colonic tissues [9]. One of the mechanisms described for the anti-cancer effect of GB is reactive oxygen species (ROS) suppression in cancerous cells, as well as inflammatory cytokines such as TNF-α, IL-6, iNOS, and COX-2. GB also inhibited the LPS-mediated activation of NF-κB and inflammatory cytokines in the murine macrophage cell line RAW 264.7 [31]. Furthermore, the therapeutic properties of GB (concentrations like 50 mg/day) have been studied thought viability assays in cancer cell lines, showing an inhibition in cellular proliferation [6]. In this regard, Hassan and colleagues reported the effect of GB in cellular suppression of several human types of cancer, such as breast, cervical, lung, colon, and prostate, whose main biochemical mechanism involves the methyl donor potential of GB [9]. In most cancers, methylation abnormalities are the most common cause of cancer development [32]. It exists evidence that alterations in this process may activate pro-oncogenes such as c-Myc and deactivate tumor suppressor genes, including p16; this process may exacerbate oxidative stress and promote tumor generation [32,33]. GB may also increase the levels of glutathione and S-adenosyl methionine and diminish the levels of other compounds related to cellular damage, such as homocysteine. Moreover, other mechanism of the effect of GB against cancer might be due to enhanced mitochondrial function contributing to a reversal of the Warburg effect, as it improves cytochrome c oxidase activity and mitochondrial respiration, which in turn, increases mitochondrial membrane potential, cellular energy levels, and generates excessive reactive oxygen species (ROS), leading to cancerous cell death [34].

## 4. Materials and Methods

### 4.1. Type of Study

This research is classified as a descriptive, quantitative, and exploratory study. Cell morphology and viability, gene expression, and protein quantification of p53 and caspase-3 were demonstrated as dependent variables, whereas GB (at specific concentrations to treat HT-29 cells) was considered independent.

### 4.2. HT-29 Cell Culture

GB assays were performed in the epithelial cell line HT-29 (ATCC^®^ 15707), a human colorectal adenocarcinoma cell line originally obtained in 1972 from a 44-year-old Caucasian female of blood group A and Rh-positive [18]. HT-29 cells were grown in Roswell Park Memorial Institute-1640 (RPMI, Corning, Manassas, VA, USA) culture medium supplemented with 10% fetal bovine serum (FBS, Corning, CA, USA), 1 mM sodium pyruvate, 0.1 mM L-glutamine (Gibco, Grand Island, NY, USA), 100 U/mL penicillin, and 100 μg/mL streptomycin, 0.25 μg/mL Amphotericin B (Antibiotic-Antimycotic solution, Caisson, Smithfield, UT, USA) and incubated at 37 °C, 95% humidity, and in an atmosphere of 5% CO_2_. Assays were performed using cells from not more than 20 passages. Treatments were with GB at various concentrations, and the determination of mRNA and proteins were performed in triplicate with three independent experiments.

### 4.3. Proliferation Assays

HT-29 cells were treated with GB (Betaine BioUltra ≥ 99.0%, Sigma-Aldrich, Darmstadt, Germany) at concentrations ranging from 0.25 mg/mL to 500 mg/mL. We used the colorimetric MTT (Sigma-Aldrich, St. Louis, MO, USA) reduction assay to determine cell viability. In brief, HT-29 cells (15,000 cells/mL per well in a 96-well plate) had their culture medium removed and were washed twice with PBS. Next, 10 µL of MTT (5 mg/mL final concentration) was added to each well and incubated for 3 h at 37 °C, in 95% humidity, and in a 5% CO_2_ atmosphere. MTT was then removed and 100 µL/well of acidified isopropyl alcohol was added until the formazan crystals dissolved at room temperature. The plate was incubated for 15 min at room temperature, and O.D.s were determined in a microplate reader (Thermo Fisher Scientific, Waltham, MA, USA) at wavelengths of 570 nm and 630 nm. As a result of the viability assays, GB concentrations for subsequent experiments were established as 5 mg/mL, 15.6 mg/mL, 31.2 mg/mL, and 62.5 mg/mL.

### 4.4. RNA Purification and cDNA Synthesis

Total RNA was purified from HT-29 cells treated with GB at 5 mg/mL, 15.6 mg/mL, 31.2 mg/mL, and 62.5 mg/mL, by the Trizol method, following the manufacturer’s instructions (Life Technologies, Carlsbad, CA, USA). RNA concentration and quality were assessed using a Nanodrop spectrophotometer, and integrity was evaluated by 1% agarose gel electrophoresis. Next, single-stranded cDNA was synthesized using 1000 ng of total RNA in a final volume of 20 mL, using a RevertAid H Minus First Strand cDNA Synthesis Kit (Thermo Fisher Scientific, Waltham, MA, USA) and oligo dT. The reaction mixture was incubated at 25 °C for 10 min, at 42 °C for 60 min, and at 70 °C for 10 min. The cDNA product of reverse transcription was stored at −80 °C, until used as a template in qPCR reactions.

### 4.5. Relative Gene Expression of p53 and Caspase-3

cDNA from HT-29 cells treated with GB was used as a template to determine the gene expression of the proliferation marker gene p53 (AB082923.1 *Homo sapiens* mRNA for p53) and the apoptosis for the gene-specific effector caspase-3 (BC016926.2 *Homo sapiens* caspase-3). In addition, for relative quantification, all samples were normalized using GAPDH as an internal reference gene. Gene amplification was performed using the Maxima SYBR Green/Rox qPCR Master Mix kit (Thermo Fisher Scientific, Waltham, MA, USA). Reactions were performed using the quantitative PCR (qPCR) method. For each reaction, 1 µL of cDNA was used as a template, plus 5 µL of the SYBR green mix (2.5 mM MgCl_2_), 1 µL of sense primer (10 mM), 1 µL of antisense primer (10 mM), and 2 µL of H_2_O. The final reaction volume was 10 µL. The primer sequences are shown in Table 1. Amplification was performed using the QuantStudio Real-Time PCR Systems (Applied Biosystems, Waltham, MA, USA), under the following conditions: initial denaturation at 94 °C for 5 min, 40 cycles of 94 °C for 30 s, 60 °C for 30 s, 72 °C for 30 s, and a final extension of 72 °C for 5 min. The relative expression was calculated using the 2^−ΔΔCt^ method. For each gene, we calculated the ΔCt (ΔCt of the gene of interest—ΔCt of the reference gene GAPDH); subsequently, the ΔΔCt was calculated by subtracting the ΔCt of a sample—ΔCt of the untreated control, later this value will be normalized using the 2^−ΔΔCt^ formula [35].

### 4.6. Determination of p53 and Caspase-3 Protein Levels

To determine the protein levels of p53 in cells treated with GB, a human p53 ELISA kit (Cat BMS256) was used, whereas the protein levels of caspase-3 were determined with the Human Caspase 3 Instant ELISA Kit (eBioscience, San Diego, CA, USA), following the manufacturer’s instructions. O.D’s were read in a Varioskcan spectrophotometer (Thermo Fisher Scientific, Waltham, MA, USA) at 450 nm as the primary wavelength and 620 nm as the reference wavelength.

### 4.7. Statistical Analysis

Data represents the mean ± SD of triplicate determinations from three independent experiments. Statistical analysis was performed using GraphPad Prism 10 (GraphPad Software Inc., San Diego, CA, USA) statistics software, first analyzing the normality of the data to determine the appropriate statistical test with the Shapiro–Wilk and Kolmogorov–Smirnov test. Once the normality of the data was confirmed, a one-way ANOVA test was performed, with a confidence level of *p* < 0.05.

## 5. Conclusions

Our study demonstrated the effect of exogenous GB in the human colorectal adenocarcinoma cell line HT-29. We initially showed a reduction in cellular proliferation by GB at concentrations of 62.5 mg/mL or higher. GB at 62.5 mg/mL was also associated with increased p53 and caspase-3 expression levels, which are related to cellular apoptosis. Therefore, exogenous GB induces apoptosis via p53 and caspase-3 in HT-29 cells in a dose-dependent manner. Although more studies are needed, adequate supplementation with GB may potentially be an adjuvant in the treatment of colorectal cancer.

## Figures and Tables

**Table 1:** Primers sequences.

Gene	Primers (5′–3′)	Amplification Size (pb)	Reference
p53	Fwd: ATCTACTGGGACGGAACAGC	136	[36]
	Rev: GTGAGGCTCCCCTTTCTTG		
Caspase-3	Fwd: CCTGGTTCATCCAGTCGCTT	100	[37]
	Rev: TCTGTTGCCACCTTTCGGTT		
GAPDH	Fwd: CTGCACCACCAACTGCTTAGC	180	[37]
	Rev: TCATGTTCTGGAGAGCCCCG		

## Data Availability

The raw data supporting the conclusions of this article will be made available by the authors on request.

## Abbreviations

The following abbreviations are used in this manuscript:

GB	Glycine betaine
ELISA	Enzyme-linked immunosorbent assay
RNA	Ribonucleic acid
cDNA	Complementary DNA (Deoxyribonucleic acid)
CRC	Colorectal cancer
ESPEN	European Society for Clinical Nutrition and Metabolism
MTT	3-(4,5-dimethylthiazol-2-yl)-2,5-diphenyl-2H-tetrazolium bromide
O.D.	Optical density

## References

[1] Rosas-Rodríguez J.A., Valenzuela-Soto E.M. (2021). The glycine betaine role in neurodegenerative, cardiovascular, hepatic, and renal diseases: Insights into disease and dysfunction networks. Life Sci..

[2] Sun J., Wen S., Zhou J., Ding S. (2017). Association between malnutrition and hyperhomocysteine in Alzheimer’s disease patients and diet intervention of betaine. J. Clin. Lab. Anal..

[3] Zhao G., He F., Wu C., Li P., Li N., Deng J., Zhu G., Ren W., Peng Y. (2018). Betaine in Inflammation: Mechanistic Aspects and Applications. Front. Immunol..

[4] Dobrijevic D., Pastor K., Nastic N., Ozogul F., Krulj J., Kokic B., Bartkiene E., Rocha J.M., Kojic J. (2023). Betaine as a Functional Ingredient: Metabolism, Health-Promoting Attributes, Food Sources, Applications and Analysis Methods. Molecules.

[5] Xu X., Gammon M.D., Zeisel S.H., Bradshaw P.T., Wetmur J.G., Teitelbaum S.L., Neugut A.I., Santella R.M., Chen J. (2009). High intakes of choline and betaine reduce breast cancer mortality in a population-based study. FASEB J..

[6] Kar F., Hacioglu C., Kacar S., Sahinturk V., Kanbak G. (2019). Betaine suppresses cell proliferation by increasing oxidative stress-mediated apoptosis and inflammation in DU-145 human prostate cancer cell line. Cell Stress Chaperones.

[7] Guo Y., Xu L.S., Zhang D., Liao Y.P., Wang H.P., Lan Z.H., Guan W.J., Liu C.Q. (2015). Betaine Effects on Morphology, Proliferation, and p53-induced Apoptosis of HeLa Cervical Carcinoma Cells in Vitro. Asian Pac. J. Cancer Prev..

[8] Seyyedsalehi M.S., Rossi M., Hadji M., Rashidian H., Marzban M., Parpinel M., Fiori F., Naghibzadeh-Tahami A., Hannun Y.A., Luberto C. (2023). Dietary Choline and Betaine Intake and Risk of Colorectal Cancer in an Iranian Population. Cancers.

[9] Hassan M.S., Khalid T., Akhlaq M., Hameed A., Sharif F., Rana S., Uroos M. (2025). Therapeutic potential of betaine and its derivatives in cancer treatment: A comprehensive review. RSC Adv..

[10] Lee J.E., Giovannucci E., Fuchs C.S., Willett W.C., Zeisel S.H., Cho E. (2010). Choline and betaine intake and the risk of colorectal cancer in men. Cancer Epidemiol. Biomark. Prev..

[11] Lu M.-S., Fang Y.-J., Pan Z.-Z., Zhong X., Zheng M.-C., Chen Y.-M., Zhang C.-X. (2015). Choline and betaine intake and colorectal cancer risk in Chinese population: A case-control study. PLoS ONE.

[12] Morgan E., Arnold M., Gini A., Lorenzoni V., Cabasag C.J., Laversanne M., Vignat J., Ferlay J., Murphy N., Bray F. (2023). Global burden of colorectal cancer in 2020 and 2040: Incidence and mortality estimates from GLOBOCAN. Gut.

[13] Fadlallah H., El Masri J., Fakhereddine H., Youssef J., Chemaly C., Doughan S., Abou-Kheir W. (2024). Colorectal cancer: Recent advances in management and treatment. World J. Clin. Oncol..

[14] Smith H.G., Nilsson P.J., Shogan B.D., Harji D., Gambacorta M.A., Romano A., Brandl A., Qvortrup C. (2024). Neoadjuvant treatment of colorectal cancer: Comprehensive review. BJS Open.

[15] Rovesti G., Valoriani F., Rimini M., Bardasi C., Ballarin R., Di Benedetto F., Menozzi R., Dominici M., Spallanzani A. (2021). Clinical Implications of Malnutrition in the Management of Patients with Pancreatic Cancer: Introducing the Concept of the Nutritional Oncology Board. Nutrients.

[16] Muscaritoli M., Arends J., Bachmann P., Baracos V., Barthelemy N., Bertz H., Bozzetti F., Hütterer E., Isenring E., Kaasa S. (2021). ESPEN practical guideline: Clinical Nutrition in cancer. Clin. Nutr..

[17] Jayalakshmi M., Vanitha V. (2017). Betaine supplementation for various clinical disorders. Asian J. Pharm. Clin. Res..

[18] Nikrandt G., Chmurzynska A. (2024). Decoding Betaine: A Critical Analysis of Therapeutic Potential Compared with Marketing Hype—A Narrative Review. J. Nutr..

[19] Wawryk-Gawda E., Chylińska-Wrzos P., Lis-Sochocka M., Chłapek K., Bulak K., Jędrych M., Jodłowska-Jędrych B. (2014). P53 protein in proliferation, repair and apoptosis of cells. Protoplasma.

[20] D’Onofrio N., Cacciola N.A., Martino E., Borrelli F., Fiorino F., Lombardi A., Neglia G., Balestrieri M.L., Campanile G. (2020). ROS-mediated apoptotic cell death of human colon cancer LoVo cells by milk δ-valerobetaine. Sci. Rep..

[21] Asadi M., Taghizadeh S., Kaviani E., Vakili O., Taheri-Anganeh M., Tahamtan M., Savardashtaki A. (2022). Caspase-3: Structure, function, and biotechnological aspects. Biotechnol. Appl. Biochem..

[22] Youn J., Cho E., Lee J.E. (2019). Association of choline and betaine levels with cancer incidence and survival: A meta-analysis. Clin. Nutr..

[23] Bostrom B., Sweta B., James S.J. (2015). Betaine for patients with acute lymphoblastic leukemia intolerant of maintenance chemotherapy due deficiency of S-adenosyl methionine. Blood.

[24] Yu J., Laybutt D.R., Youngson N.A., Morris M.J. (2022). Concurrent betaine administration enhances exercise-induced improvements to glucose handling in obese mice. Nutr. Metab. Cardiovasc. Dis..

[25] Lee E.J., An D., Nguyen C.T., Patil B.S., Kim J., Yoo K.S. (2014). Betalain and betaine composition of greenhouse-or field-produced beetroot (*Beta vulgaris* L.) and inhibition of HepG2 cell proliferation. J. Agric. Food Chem..

[26] Kulthanaamondhita P., Kornsuthisopon C., Chansaenroj A., Phattarataratip E., Sappayatosok K., Samaranayake L., Osathanon T. (2024). Betaine Induces Apoptosis and Inhibits Invasion in OSCC Cell Lines. Int. J. Mol. Sci..

[27] Liebl M.C., Hofmann T.G. (2021). The Role of p53 Signaling in Colorectal Cancer. Cancers.

[28] Nowacki L., Vigneron P., Rotellini L., Cazzola H., Merlier F., Prost E., Ralanairina R., Gadonna J.P., Rossi C., Vayssade M. (2015). Betanin-enriched red beetroot (*Beta vulgaris* L.) extract induces apoptosis and autophagic cell death in MCF-7 cells. Phytother. Res..

[29] Chávez A.H., Alanis A.G., Flores R.G., Guerra P.T., Villalobos J.M.R., Santibañez K.S.M., Padilla C.R., Ochoa G.G., Vasquez A.O., Sáenz C.I.R. (2023). In vitro additive effect of Chlorella sorokiniana in combination with Vincristine on HT-29 colon cancer cells growth inhibition. Nutr. Clín. Diet. Hosp..

[30] Liu L., Yan Q., Chen Z., Wei X., Li L., Tang D., Tan J., Xu C., Yu C., Lai Y. (2023). Overview of research progress and application of experimental models of colorectal cancer. Front. Pharmacol..

[31] Kim D.H., Sung B., Kang Y.J., Jang J.Y., Hwang S.Y., Lee Y., Kim M., Im E., Yoon J.-H., Kim C.M. (2014). Anti-inflammatory effects of betaine on AOM/DSS--induced colon tumorigenesis in ICR male mice. Int. J. Oncol..

[32] Dong Y., Zhao H., Li H., Li X., Yang S. (2014). DNA methylation as an early diagnostic marker of cancer. Biomed. Rep..

[33] Gu M., Ren B., Fang Y., Ren J., Liu X., Wang X., Zhou F., Xiao R., Luo X., You L. (2024). Epigenetic regulation in cancer. MedComm.

[34] Lee I. (2015). Betaine is a positive regulator of mitochondrial respiration. Biochem. Biophys. Res. Commun..

[35] Livak K.J., Schmittgen T.D. (2001). Analysis of relative gene expression data using real-time quantitative PCR and the 2^−ΔΔCT^ method. Methods.

[36] Zhang P., Suidasari S., Hasegawa T., Yanaka N., Kato N. (2014). Vitamin B_6_ activates p53 and elevates p21 gene expression in cancer cells and the mouse colon. Oncol. Rep..

[37] Stephens-Camacho N., Rodríguez J.A.R., Islas-Zamorano A.P., Magaña-Gómez J.A., Flores-Mendoza L.K. (2022). La sucralosa promueve la polarización a macrófagos proinflamatorios M1. Rev. Chil. Nutr..

